# Sugar and acid profile of loquat (*Eriobotrya japonica* Lindl.), enzymes assay and expression profiling of their metabolism-related genes as influenced by exogenously applied boron

**DOI:** 10.3389/fpls.2022.1039360

**Published:** 2022-10-20

**Authors:** Muhammad Moaaz Ali, Raheel Anwar, Rana Naveed Ur Rehman, Shaghef Ejaz, Sajid Ali, Ahmed F. Yousef, Sezai Ercisli, Xiaobo Hu, Youming Hou, Faxing Chen

**Affiliations:** ^1^ State Key Laboratory of Ecological Pest Control for Fujian and Taiwan Crops, College of Plant Protection, Fujian Agriculture and Forestry University, Fuzhou, China; ^2^ Institute of Subtropical Fruits, Fujian Agriculture and Forestry University, Fuzhou, China; ^3^ Institute of Horticultural Sciences, University of Agriculture, Faisalabad, Pakistan; ^4^ Department of Horticulture, Faculty of Food and Crop Science, Pir Mehr Ali Shah (PMAS)-Arid Agriculture University, Rawalpindi, Pakistan; ^5^ Department of Horticulture, Faculty of Agricultural Sciences and Technology, Bahauddin Zakariya University, Multan, Pakistan; ^6^ Department of Horticulture, College of Agriculture, University of Al-Azhar (Branch Assiut), Assiut, Egypt; ^7^ Department of Horticulture, Agricultural Faculty, Ataturk University, Erzurum, Turkey

**Keywords:** malic acid, PEPC, fruit quality, malate dehydrogenase, borax, fructokinase, sucrose, liquid chromatography

## Abstract

Soluble sugars and organic acids are the most abundant components in ripe fruits, and they play critical roles in the development of fruit flavor and taste. Some loquat cultivars have high acid content which seriously affect the quality of fruit and reduce the value of commodity. Consequently, studying the physiological mechanism of sugar-acid metabolism in loquat can clarify the mechanism of their formation, accumulation and degradation in the fruit. Minerals application has been reported as a promising way to improve sugar-acid balance of the fruits. In this study, loquat trees were foliar sprayed with 0.1, 0.2 and 0.3% borax, and changes in soluble sugars and organic acids were recorded. The contents of soluble sugars and organic acids were determined using HPLC-RID and UPLC-MS, respectively. The activities of enzymes responsible for the metabolism of sugars and acids were quantified and expressions of related genes were determined using quantitative real-time PCR. The results revealed that 0.2% borax was a promising treatment among other B applications for the increased levels of soluble sugars and decreased acid contents in loquats. Correlation analysis showed that the enzymes i.e., SPS, SS, FK, and HK were may be involved in the regulation of fructose and glucose metabolism in the fruit pulp of loquat. While the activity of NADP-ME showed negative and NAD-MDH showed a positive correlation with malic acid content. Meanwhile, *EjSPS1*, *EjSPS3*, *EjSS3*, *EjHK1*, *EjHK3*, *EjFK1*, *EjFK2*, *EjFK5*, and *EjFK6* may play an important role in soluble sugars metabolism in fruit pulp of loquat. Similarly, *EjPEPC2*, *EjPEPC3*, *EjNAD-ME1*, *EjNAD-MDH1*, *EjNAD-MDH5-8*, *EjNAD-MDH10*, and *EjNAD-MDH13* may have a vital contribution to malic acid biosynthesis in loquat fruits. This study provides new insights for future elucidation of key mechanisms regulating soluble sugars and malic acid biosynthesis in loquats.

## Introduction

Fruits have their own biochemical and nutritional features over the course of their growth, which eventually results in their distinct fruit quality (Bermejo and Cano, 2012; Ren et al., 2015; Zhang et al., 2021). This process promotes the development of sugar and organic acid metabolites, which are important for the development of fruit flavor since the growth of fruit is usually accompanied by the accumulation and degradation of sugars and organic acids (; Zhang et al., 2014: 2021). The balance between the sugar-acid production, breakdown, and vacuole storage determines their ultimate content in ripe fruits (Ruffner et al., 1984; Pan et al., 2021). Fruits are categorised into three major categories based on the amount of organic acid they contain: malic acid, citric acid, and tartaric acid (Batista-Silva et al., 2018). Malic acid is the primary organic acid type in loquat (Li et al., 2015b; Ali et al., 2021a).

Loquat (*Eriobotrya japonica* Lindl.) is an evergreen fruit tree native to China (Ali et al., 2021b). It is a member of family Rosaceae, subfamily Maloideae (Ali et al., 2021c). Vitamin A, vitamin B6, potassium, magnesium, and dietary fibre are all abundant in this fruit (Badenes et al., 2003). It is an orange-colored fruit with a mildly sweet flavor (Zhi et al., 2021). Loquat fruit cannot be preserved for lengthy periods of time due to its soft and juicy flesh and thin peel (Tian et al., 2011). Besides its utilization as fruit, it is a good source of honey. Its flowers are much attractive to honey bees, especially white-colored flowers (Karadeniz et al., 2012). Japan, Korea, India, Pakistan, and China’s south-central area are the main producers of loquat (Ali et al., 2021d). In California, it’s also cultivated as an ornamental shrub (LaRue, 2020). Loquat is grown on more than 130 thousand hectares in China, making it the world’s biggest producer and exporter. China produces 650 thousand tonnes of loquats each year (Zheng et al., 2019). High fruit acidity and low sugars have been major factors lowering fruit quality and commodity value in commercial loquat production (Chen et al., 2009).

The mineral elements are absorbed to variable degrees and play important roles in fruit quality, since many of them are required for photosynthesis, respiration, energy metabolism, and cell structure (Broadley et al., 2012; Engels et al., 2012; Wiesler, 2012; Ali et al., 2021d). In comparison to soil application, foliar application of nutrients has a 10-20 percent greater influence (Zaman and Schumann, 2006; Ali et al., 2021b). Boron (B) is involved in a variety of metabolic functions, such as sugar transport and respiration (Ali et al., 2019), cell wall formation (Brown et al., 2002), cell division and elongation (Goldbach et al., 2001; de Oliveira et al., 2006), membrane stability, carbohydrate metabolism and Ca^2+^ uptake, hormone activation, root development, water translocation (Zhao and Oosterhuis, 2002; Sheng et al., 2009), and the activation of dehydrogenase enzymes (Marschner, 1995; El-Sheikh et al., 2007). However, the physiological and molecular functions of boron in regulating sugar-acid metabolism are not fully known at this time, and more research is required to clarify this.

Here, in this study, loquat trees were foliar sprayed with 0.1, 0.2 and 0.3% borax, and changes in soluble sugars and organic acids were recorded. Soluble sugars (fructose, glucose and sucrose) and organic acids (fumaric acid, ascorbic acid, malic acid, *cis*-aconitic acid and acetic acid) were quantified using HPLC and UPLC, respectively. The HPLC method can directly determine oligosaccharide with a simple sample preparation. It is one of the most promising methods for sugar analysis, due to its universality, time efficiency, accuracy, and selectivity for the quantification of carbohydrates (Kakita et al., 2002). Similarly, UPLC method is used to determine organic acid content of fruits, because of its simplicity, speed and stability (Fedorova et al., 2020). We not only investigated the effect of different concentrations of B on yet unexplored aspects of loquat sugar-acid metabolism but also segregated concentration-dependent variations in activities of related enzymes and relative expression of their biosynthesis-related genes.

## Materials and methods

### Plant material, experimental design and treatments

The young loquat trees (Cv. Jiefangzhong), growing in an orchard located in the subtropical area of Fujian province (Fuqing) (25°47’26.0”N 119°20’31.0”E), were selected for this study. The loquat trees ranged in height from 4 to 5.5 m and had a canopy diameter of 4 to 5 m. The spacing between each tree in the planting was roughly 6 × 6 m. Throughout the last three growing seasons, loquat trees have been subjected to methodical pruning and thinning, as well as fertilization with nitrogen (N), phosphorus (P) and potassium (K) (15:15:15) at a rate of 5 kg per plant every season. A randomized block design was used to allocate the distribution of plants for various treatments (RCBD). Each treatment had a total of four replications, or blocks, allocated to it, and each individual tree was counted as a single replicate for each treatment. Standard agricultural procedures were used throughout the production of loquats. These activities included drip irrigation, mineral supplementation, weed management, and the management of insects/pests and diseases. The experiment consisted of four separate treatments, the control (water spray), 0.1% borax, 0.2% borax, and 0.3% borax respectively. These foliar concentrations were chosen after an earlier study about phyto-nutritional composition of loquat (Ali et al., 2021b). The foliar treatment was done twice during the full bloom stage (the first week of January, 2020), with a three-week break in between each application. Early in the morning, a foliar spray of B was applied to loquat trees using an electronic sprayer with a capacity of 5 L that was set at a constant pace. Fully ripened fruits were sampled from the sun-exposed tree canopy (Ali et al., 2021c; Ali et al., 2021b), at about 1.5–2.5 m height, 90 days after first foliar spray, and brought back immediately to the laboratory (Institute of Tropical and Subtropical Fruits, FAFU).

### Fruit weight and size

The average fresh weight, length (from the tallest point), and width (from the widest point) of the fruit were determined by averaging five batches of fruit, each of which consisted of 10 loquats from the same treatment. The fruit’s weight was measured using a digital weighing balance (MJ-W176P, Panasonic, Japan), and its length and diameter were gauged with digital Vernier callipers (DR-MV0100NG, Ningbo Dongrun Imp. & Exp. Co., Ltd., China). The length-to-width ratio, hereafter referred to as the fruit shape index, was determined by dividing each fruit’s length by its diameter.

### Soluble solids, titratable acids, sugar-acid ratio and fruit juice pH

Using a titrimetric approach based on NaOH (Hortwitz, 1960), the total titratable acids were calculated and shown as a percentage of citric acid. A portable digital refractometer was used to calculate the total soluble solids (Atago, Hybrid PAL-BXIACID F5, Japan). Total soluble solids in the sample were divided by total titratable acids to get the sugar-acid ratio. The acidity level of fruit juice was measured using a digital pH meter (Hanna, HI-98107, Mauritius).

### Soluble sugars determination through HPLC-RID

The contents of soluble sugars were determined through high-performance liquid chromatography – refractive index detection (HPLC-RID) as earlier described by Yu et al. (2021). Fruit samples (pulp stored at -80°C) were ground up in liquid nitrogen, and the resulting 2 g of fine powder was mixed with a modest quantity of polyvinylpyrrolidone in 10 mL of 95% methanol. The supernatant fluid was collected after ultra-sonification at 40°C for 30 min and centrifugation at 1000 rpm for 10 min. With 8 mL of ultrapure water, the procedure was repeated using the leftover residue. A 0.22 m syringe filter was then used to filter the clear liquid (ANPEL, China). A Waters 2695 autosampler system was used for HPLC-RID analysis. Ellistat Supersil NH_2_ column (4.6 mm × 250 mm, 5 µm particle size) (Waters Inc, Zellik, Belgium) was used to separate soluble sugars, operated at 40°C. The mobile phases consisted of 82% acetonitrile and 18% ultrapure water solution mixture. The amount of the injection was 20 µL, and the flow rate was 1.2 mL per min. In the end, the concentration of each and every solitary soluble sugar was determined by using the calibration curve of the standard that corresponded to it. The standards of fructose (99%), glucose (99.5%)) and sucrose (99.5%) were obtained from Sigma-Aldrich, USA. Every single one of the assays for soluble sugars was carried out using three separate samples. The output was given in milligrams per milliliter, which was denoted with the notation mg·ml^-1^.

### Organic acids determination through UPLC-MS

The extraction of organic acids was carried out using the method outlined by Nour et al. (2010), although with minor adjustments. In order to extract juice from the loquat fruits, they were first halved and then pressed. After going through three layers of gauze material, the pulp was removed. Following centrifugation of the juice at 4000 rpm for 15 min, the supernatant was diluted 25 times and passed through an MF-Millipore™ Membrane Filter with a pore size of 0.22 μm in diameter. The ultra-performance liquid chromatography – mass spectrometry (UPLC-MS) technique was used in order to investigate organic acids. A sample of 10 µL of eluate was injected into an Acquity UPLC HSS T3 column (1.8 µm particle size, 2.1 mm × 100 mm). When employing a solution containing 0.025% H_3_PO_4_ as the solvent, the flow rate was 0.2 mL per min. Organic acids were detected at 210 nm, while column temperature was 30°C. A Waters 2996 diode array detector (Waters Corporation, USA) was used to detected the eluted peaks. Using the calibration curve of the relevant standard, the contents of the various organic acids were able to be determined and computed. The standards of fumaric acid (99%), ascorbic acid (99%), malic acid (99%), *cis*-aconitic acid (98%) and acetic acid (99.7%) were obtained from Sigma-Aldrich, USA. Every one of the assays for organic acids was carried out using three separate samples. The output was reported in milligrams per milliliter of fresh juice, which is abbreviated as mg·ml^-1^ juice.

Limits of detection and quantification were included as parts of the HPLC-RID and UPLC-MS procedures’ validation parameters (Ribani et al., 2004). The peaks were identified by their retention times, comparing the UV–Visible spectra and spiking with standards. Quantification has been done using an external standard curve with five points (**Table 1**; **Figures S1, S2**).

**Table 1:** Validation parameters for HPLC/UPLC method.

Sugar/Acid type	Linearity (R^2^)	Standard deviation (SD)	Slope (y)	Response (Sy)	Sy/y	LOD* (mg·ml^-1^)	LOQ** (mg·ml^-1^)
Fructose	0.9767	1.5811	6544.8	1845.64	0.28	0.93	2.82
Glucose	0.9864	1.5811	5743.4	1231.92	0.21	0.71	2.15
Sucrose	0.9799	1.5811	4413.2	1154.19	0.26	0.86	2.62
Fumaric acid	0.9713	0.0016	1814350	381.7	0.0002	0.0006	0.0021
Ascorbic acid	0.9804	0.0174	232772.1	1303.62	0.0056	0.0184	0.056
Malic acid	0.9998	0.3488	31444.57	626.07	0.0199	0.0657	0.1991
*Cis*-aconitic acid	0.9777	0.0014	669100	228.85	0.0003	0.0011	0.0034
Acetic acid	0.9771	0.3488	14109.31	820.78	0.0581	0.1919	0.5817

*Limit of detection; **Limit of quantification

### Enzymes extraction and activity assay

The enzymes responsible for sugar [sucrose-phosphate synthase (SPS), sucrose synthase (SS), hexokinase (HK) and fructokinase (FK)] and acid metabolism [phospho*enol*pyruvate carboxylase (PEPC), NADP – dependent malic enzyme (NADP-ME) and NAD – malate dehydrogenase (NAD-MDH)] were extracted and measured using the Solarbio enzyme activity kits (Solarbio Life Sciences, Beijing, China) according to the manufacturer’s instructions (Zhang et al., 2021). The extraction kits were based on the earlier determined methods for SPS (Schrader and Sauter, 2002), SS (Schrader and Sauter, 2002), HK (Pancera et al., 2006), FK (Papagianni and Avramidis, 2011), PEPC (Zhang et al., 2008), NADP-ME (Spampinato et al., 1994), and NAD-MDH (Yao et al., 2011).

### RNA extraction and real-time quantitative PCR

Total RNA was extracted from loquat fruit pulp using a Total RNA kit (TianGen Biotech, Beijing, P.R. China). NanoDrop N-1000 spectrophotometer (NanoDrop technologies, Wilmington, DE, USA) was used to analyze RNA concentration and purity. First-strand cDNA was synthesized from 1 µg of total RNA using the Prime Script RT Reagent Kit with a gDNA Eraser (TaKaRa, Dalian, China). High-performance real-time PCR (LightCycler^®^ 96, Roche Applied Science, Penzberg, Germany) was used for the qPCR analysis. Primers used in quantitative real-time polymerase chain reaction (qRT-PCR) are included in **Table S1**, which were designed using Primer-blast.

The reaction mixture contained 10 μL 2×RealStar Green Fast Mixture (GenStar, Bejing, China), 1 µL cDNA, 0.25 µM of each primer and water was added to make a final volume of 20 µL. The qRT-PCR protocol started with a 5 min “preincubation” at 95°C, then 40 cycles at 95°C for 10 s and 60°C for 30 s, a “melting” step at 95°C for 10 s, 65°C for 1 min, and 97°C for 1 s, and a “cooling” phase at 37°C for 30 s. The 2^-ΔΔct^ approach (Munhoz et al., 2015) was used to determine relative gene expression, with the actin protein (EVM0004523.1) serving as the internal control (Gan et al., 2020). The validation of 2^−ΔΔCt^ method was carried out by ΔCt variation analysis at different template concentrations (Livak and Schmittgen, 2001; Xu et al., 2017; De Rossi et al., 2021). Each sample was analysed using three biological replicates.

### Statistical analysis

The collected data was analyzed using an ANOVA with the help of the statistical programme “Statistix 8.1” (https://www.statistix.com/). Means of replicated data from each treatment were compared using Fisher’s least significant difference (LSD) method, when *p ≤ 0.05*. The Pearson (n) approach was used in ‘Statistix 8.1’ to calculate the correlation coefficient values, and “TBtools ver. 0.6655” (https://github.com/CJ-Chen/TBtools) was used to depict the data as a heat map. Principle component analysis (PCA) of treatments and tested variable was done through Pearson (*n*) method using “XLSTAT ver. 2019” (https://www.xlstat.com/en/).

## Results

### Fruit weight and size

**Figure 1:**
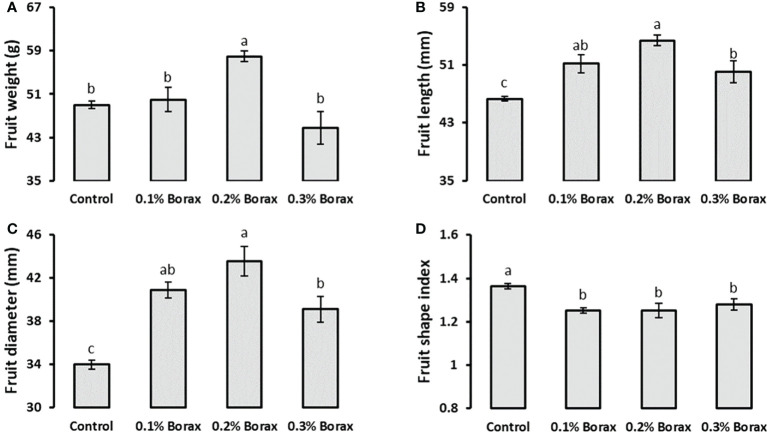
Effect of foliar application of B on weight **(A)**, length **(B)**, diameter **(C)**, and shape index **(D)** of loquat fruits. Loquat plants were foliar sprayed with B twice at blooming stage. Same letters indicate non-significant difference among treatments according to Fisher’s least significant difference (LSD) test, when *p ≤ 0.05*. Vertical bars indicate mean ± standard error (n=4, 4-block RCBD arrangement).

Loquat plants treated with foliar supplied B exhibited a significant increase (*p ≤ 0.05*) in fruit weight and size (fruit length, width, and fruit shape index) as compared to untreated plants. The plants receiving 0.2% borax exhibited maximum fruit weight (57.95 g), which was 18.19% higher than that of untreated plants (**Figure 1A**). Regardless of concentration, B improved the loquat fruit length by 8-18% as compared to control (**Figure 1B**). The maximum fruit width (43.52 mm) was recorded in the plants treated with 0.2% borax followed by the plants receiving 0.1% (40.88 mm) and 0.3% borax (39.11 mm) (**Figure 1C**). Boron application reduced fruit shape index regardless of concentration applied, indicating its possible role in improving fruit size in terms of diameter. The minimum fruit shaped index (1.25) was recorded in the plants treated with 0.1-0.2% borax, which was 8% less as compared to that of untreated plants (**Figure 1D**).

### Soluble solids, titratable acids, sugar-acid ratio and fruit juice pH

**Figure 2:**
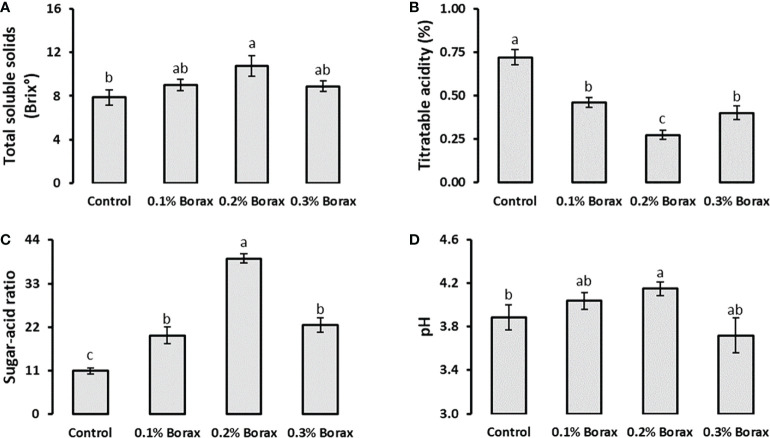
Effect of foliar application of B on total soluble solids **(A)**, titratable acidity **(B)**, sugar-acid ratio **(C)**, and pH **(D)** of loquat fruits. Same letters indicate non-significant difference among treatments according to Fisher’s least significant difference (LSD) test, when *p ≤ 0.05*. Vertical bars indicate mean ± standard error (n=4, 4-block RCBD arrangement).

Total soluble solids (TSS) and titratable acidity (TTA) of loquat fruits showed reciprocal responses to each other. Foliar application of 0.2% borax enhanced TSS by 36.86%, while reduced the TTA by 61.90% comparing with control (**Figures 2A, B**). The plants receiving foliar application of 0.2% B exhibited 3.60-fold increase in sugar-acid ratio, as compared to control. The increased sugar-acid ratio and fruit juice pH indicates the positive influence of applied treatments on sugars accumulation in loquat fruits (**Figures 2C, D**).

### Soluble sugars

**Figure 3:**
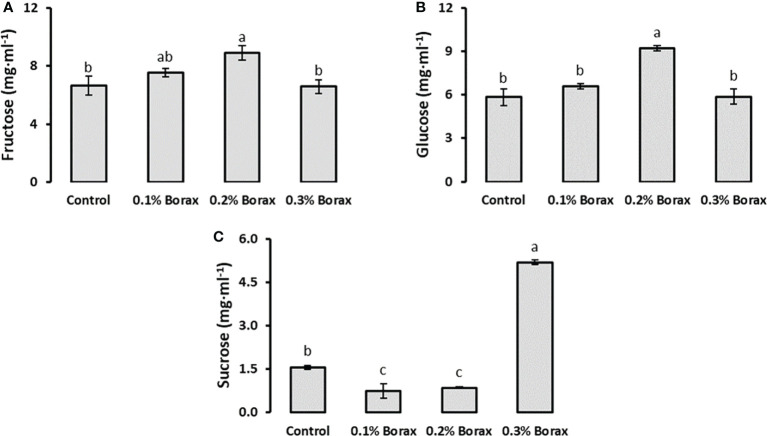
Effect of foliar application of B on soluble sugars i.e., fructose **(A)**, glucose **(B)**, and sucrose **(C)** content of loquat fruits. Same letters indicate non-significant difference among treatments according to Fisher’s least significant difference (LSD) test, when *p ≤ 0.05*. Vertical bars indicate mean ± standard error (n=4, 4-block RCBD arrangement).

Three soluble sugars i.e., fructose, glucose and sucrose were quantified in the fruit pulp of B-treated loquat fruits through HPLC (**Figure 3**). The results revealed that fructose and glucose were the abundant soluble sugars in loquat pulp as compared to sucrose contributing 45.31%, 41.95% and 12.74%, respectively. The fructose and glucose accumulation in loquat fruits showed same pattern with respect to applied treatments. The plants receiving exogenous application of 0.2% borax exhibited maximum fruit fructose level (8.91 mg·ml^-1^) among all other treatments, which was 1.34-times higher (33.89%) than that of untreated plants (**Figure 3A**). Among B treatments, 0.2% borax improved fruit glucose level by 58.21%, as compared to the fruit glucose level of untreated plants (**Figure 3B**). In case of fruit sucrose, plants receiving 0.3% borax showed a significant (*p ≤ 0.05*) improvement in sucrose level, which was recorded as 3.31-fold higher than control (**Figure 3C**).

### Organic acids

**Figure 4:**
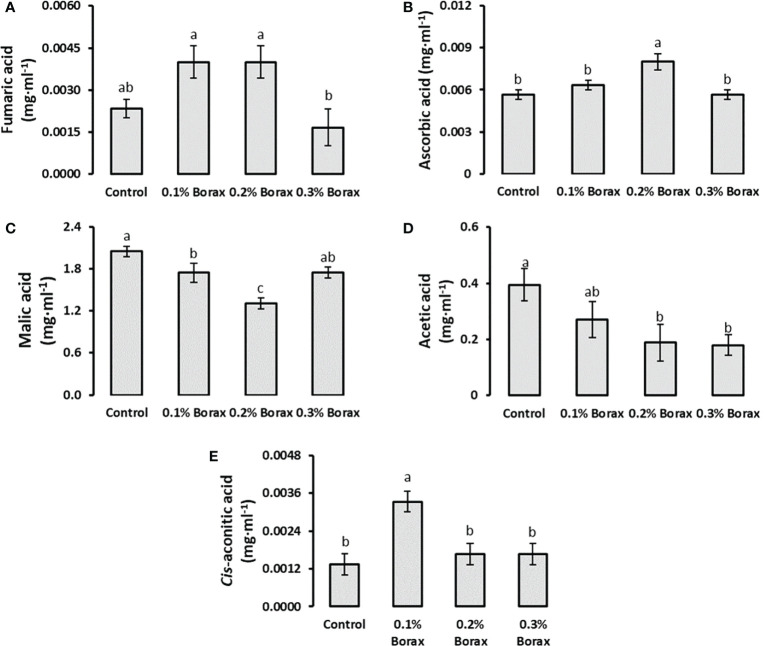
Effect of foliar application of B on the level of organic acids i.e., fumaric acid **(A)**, ascorbic acid **(B)**, malic acid Ali et al.,, acetic acid **(D)**, and *cis*-aconitic acid **(E)** in loquat fruits. Same letters indicate non-significant difference among treatments according to Fisher’s least significant difference (LSD) test, when *p ≤ 0.05*. Vertical bars indicate mean ± standard error (n=4, 4-block RCBD arrangement).

Five organic acids i.e., fumaric acid, ascorbic acid, malic acid, *cis*-aconitic acid and acetic acid were quantified in the fruit pulp of B-treated loquat fruits through UPLC. The results revealed that malic acid was the most abundant organic acid in loquat pulp followed by acetic acid contributing 81.12% and 18.21%, respectively. The proportion of fumaric acid, ascorbic acid and *cis*-aconitic acid was less than 1% among tested organic acids (**Figures 4A, B, E**). The exogenous application of B significantly reduced the malic acid concentration as compared to control, ultimately reduced overall acidity of the fruits. The plants receiving 0.2% borax exhibited minimum fruit malic acid level (1.31 mg·ml^-1^) among all other treatments, which was 1.56-times (36.07%) lower than that of untreated plants (**Figure 4C**). In case of acetic acid content, it was observed that B treatments reduced the acetic acid content in dose-dependent manner (**Figure 4D**).

### Key enzymes involved in soluble sugars metabolism

**Figure 5:**
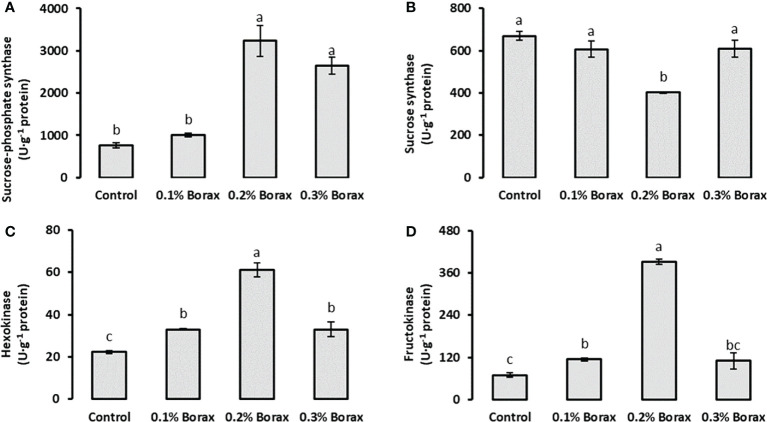
Effect of foliar application of B on the activities of key enzymes involved in soluble sugars metabolism i.e., sucrose-phosphate synthase **(A)**, sucrose synthase **(B)**, hexokinase **(C)** and fructokinase **(D)** of loquat fruits. Same letters indicate non-significant difference among treatments according to Fisher’s least significant difference (LSD) test, when *p ≤ 0.05*. Vertical bars indicate mean ± standard error (n=4, 4-block RCBD arrangement).

The SPS activity in the fruit pulp of loquat significantly (*p ≤ 0.05*) increased with B application. The maximum SPS activity was detected in the fruit pulp of the plants treated with 0.2% borax (3237.07 U·g^-1^ protein) (**Figure 5A**). Interestingly, the SS activity was found decreased with the foliar application of 0.2% borax (401.97 U·g^-1^ protein) as compared to control (669.69 U·g^-1^ protein), which was 39.98% reduced as compared to control (**Figure 5B**). Unlike SS, HK and FK activities were recorded improved in fruit pulp of loquat with all preharvest treatments of B. The maximum HK activity (61.22 U·g^-1^ protein) was measured in the fruit pulp of the plants treated with 0.2% borax (**Figure 5C**). Similarly, maximum FK activity was recorded in fruit pulp of the loquat plants receiving foliar application of 0.2% borax (391.50 U·g^-1^ protein), which was 5.61-fold higher than that of untreated plants (**Figure 5D**).

### Key enzymes involved in malic acid metabolism

**Figure 6:**
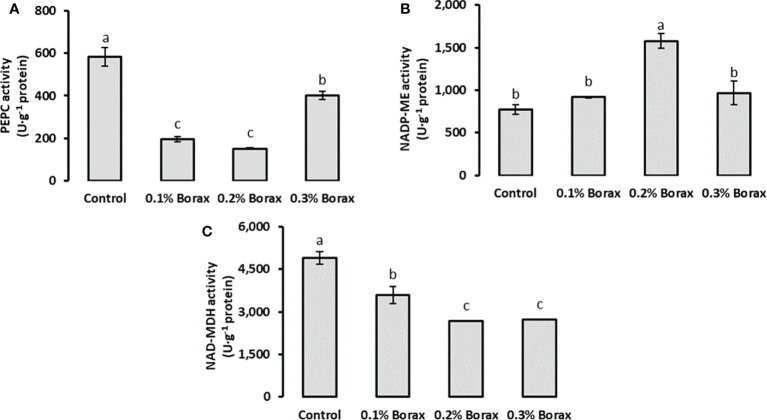
Effect of foliar application of B on the activities of key enzymes involved in malic acid metabolism i.e., PEPC **(A)**, NADP-ME **(B)**, and NAD-MDH **(C)** of loquat fruits. Same letters indicate non-significant difference among treatments according to Fisher’s least significant difference (LSD) test, when *p ≤ 0.05*. Vertical bars indicate mean ± standard error (n=4, 4-block RCBD arrangement).

The PEPC activity in the fruit pulp of loquat significantly (*p ≤ 0.05*) reduced with B application. The minimum PEPC activity was detected in the fruit pulp of the plants treated with 0.2% borax (150.87 U·g^-1^ protein) (**Figure 6A**). Interestingly, the NADP-ME activity was found increased with the foliar application of B at the concentration of 0.2%. The maximum NADP-ME activity was recorded in the fruit pulp of loquats receiving 0.2% borax (1578.76 U·g^-1^ protein) in comparison with control (771.68 U·g^-1^ protein), which were 2.04-fold higher that of untreated plants, respectively (**Figure 6B**). Conversely, the exogenous application of B significantly reduced the activity of NAD-MDH as compared to control. Among B treatments, the minimum NAD-MDH activity level was recorded in the loquats treated with 0.20-0.30% borax (2668.87-2733.18 U·g^-1^ protein) (**Figure 6C**).

### Expression profiling of soluble sugars metabolism-related genes

**Figure 7:**
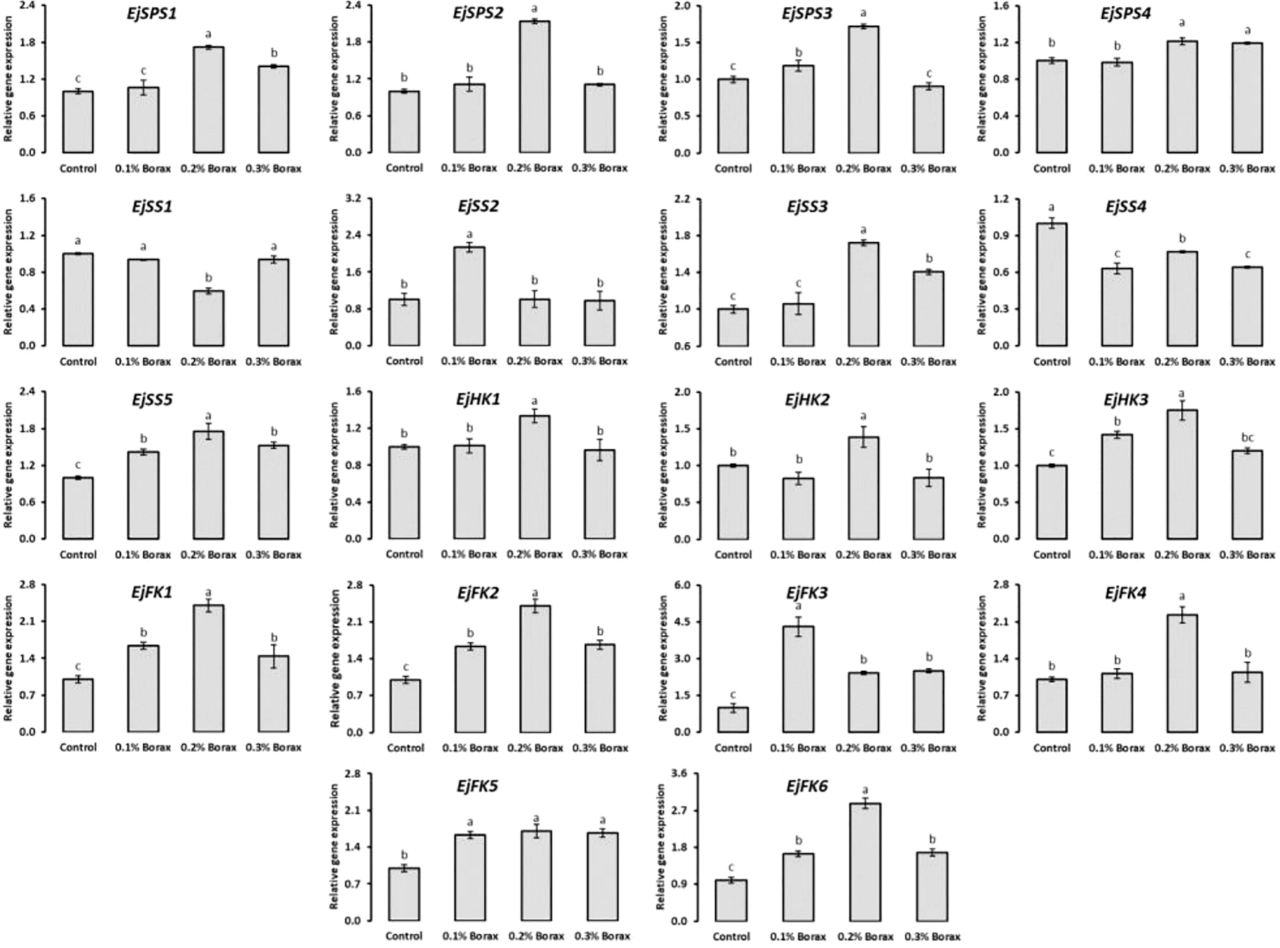
The expression profiling of core genes involved in soluble sugars metabolism of loquat as influenced by the foliar application of B Same letters indicate non-significant difference among treatments according to Fisher’s least significant difference (LSD) test, when *p ≤ 0.05*. Vertical bars indicate mean ± standard error (4 biological and 3 technical replicates).

The expression patterns of core genes i.e., *EjSPS1-4*, *EjSS1-5*, *EjHK1-3*, and *EjHK1-6* encoding key enzymes i.e., SPS, SS, HK and FK responsible for the metabolism of soluble sugars in fruit pulp of loquat were studied (**Figure 7**). The expression patterns of *EjSPS1-4* genes increased with the foliar application of B. Briefly, the relative expression of *EjSPS1* was recorded maximum in the fruit pulp of loquat when treated with 0.2% borax. Similarly, *EjSPS2* was maximally expressed under the influence of 0.2% borax, which was 2.14-fold higher than that of control. The *EjSPS3* and *EjSPS4* exhibited maximum upregulation under the influence of 0.1-0.2% and 0.2-0.3% borax, respectively.

The *EjSS3* and *EjSS5* were maximally expressed under the influence of 0.2% borax. Specifically, *EjSS1* was significantly (*p ≤ 0.05*) down-regulated by aforementioned treatment, while showed non-significant (*p ≤ 0.05*) variation under the influence of 0.1 and 0.3% borax. The *EjSS2* significantly (*p ≤ 0.05*) upregulated under the influence of 0.1% borax by 2.14-fold. The maximum expression of *EjSS3* was recorded in the loquats treated with 0.2% borax. Foliar application of B significantly (*p ≤ 0.05*) reduced the expression of *EjSS4* as compared to control. Conversely, *EjSS5* was found upregulated under the influence of B. The maximum relative expression of *EjSS5* was recorded in the loquat treated with 0.2% borax.

Among B treatments, 0.2% borax significantly (*p ≤ 0.05*) upregulated the expressions of *EjHK1-3*. The expressions of *EjHK1* and *EjHK2* remained unchanged with B treatments except when loquats received 0.20% borax. While, in the case of *EjHK3*, 0.1% borax also upregulated the expression by 41.9%.

The relative expression level of *EjFK1* was significantly (*p ≤ 0.05*) increased with the foliar application of B. The *EjFK1* was maximally upregulated under the influence of 0.2% borax (by 2.41-fold). Similarly, in the case of *EjFK2*, the maximum transcript level was observed in the loquats treated with 0.2% borax (3.43). The borax application significantly (*p ≤ 0.05*) upregulated *EjFK3* when applied at the concentration of 0.10% and reduced with increase in its concentration. Among B treatments, 0.2% borax maximally upregulated the *EjFK4* by 2.23-fold. Although the maximum expressions of *EjFK5* and *EjFK6* were recorded in the loquats treated with 0.2% borax, their levels remained upregulated with all B treatments (**Figure 7**).

### Expression profiling of malic acid metabolism-related genes

**Figure 8:**
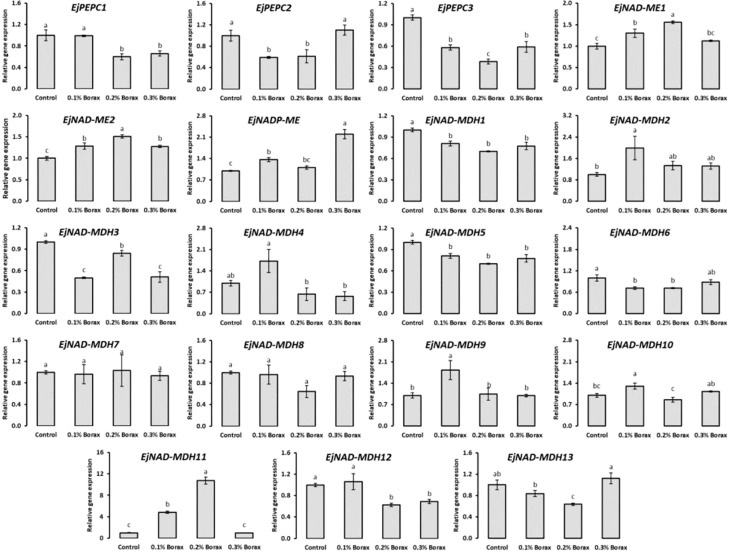
The expression profiling of core genes involved in malic acid metabolism of loquat as influenced by the foliar application of B. Same letters indicate non-significant difference among treatments according to Fisher’s least significant difference (LSD) test, when *p ≤ 0.05*. Vertical bars indicate mean ± standard error (4 biological and 3 technical replicates).

The expression patterns of core genes i.e., *EjPEPC*, *EjNAD(P)ME* and *EjNAD-MDH* encoding key enzymes i.e., PEPC, NADP-ME and NAD-MDP responsible for the malic acid metabolism in fruit pulp of loquat were studied (**Figure 8**). The expression of *EjPEPC1* found significantly decreased with the foliar application of 0.2-0.3% borax. The loquats treated with 0.1-0.2% borax exhibited downregulated expression of *EjPEPC2*. *EjPEPC2* was minimally expressed under the influence of 0.1 and 0.2% borax, which was ≥40% lower than that of control. The *EjPEPC3* also exhibited maximum downregulation under the influence of 0.2% borax.

The relative expression patterns of *EjNAD(P)-ME* genes increased with the foliar application of B. Among B treatments, 0.1-0.2% borax significantly (*p ≤ 0.05*) upregulated the expressions of *EjNAD-ME1*. Specifically, the maximum expression of *EjNAD-ME1* was recorded in the loquats treated with 0.2% borax, which were 1.56-fold higher than that of untreated loquats. Similarly, 0.1-0.3% borax significantly (*p ≤ 0.05*) improved the expression of *EjNAD-ME2* in fruit pulp. The expression of *EjNADP-ME* remained unchanged with B treatments except when loquats received 0.1 and 0.3% borax. Its maximum expression was recorded under the influence of 0.3% borax which was 2.20-fold higher than that of control loquats.

The expression of *EjNAD-MDH1* decreased with the application of borax, regardless of its concentration. The lowest *EjNAD-MDH1* transcript was recorded in the loquats treated with 0.2% borax. *EjNAD-MDH2* was maximally expressed under the influence of 0.1% borax, which was 2-fold higher than that of control. The *EjNAD-MDH3* exhibited downregulation under the influence of 0.1-0.3% borax. The relative expression pattern of *EjNAD-MDH4* comparatively increased with the foliar application of 0.1% borax, while remained unchanged under the influence of other treatments. The expression of *EjNAD-MDH5* significantly reduced with the foliar application of B, regardless of its concentration applied. The expression of *EjNAD-MDH6* reduced with the application of 0.1-0.2% borax. The minimum expression of *EjNAD-MDH6* was observed in the loquats treated with 0.2% borax, which was 29% less than that of control. The relative expression pattern of *EjNAD-MDH7* and *EjNAD-MDH8* remained unchanged under the influence of B treatments. The *EjNAD-MDH9* and *EjNAD-MDH10* exhibited its upregulated expression only under the influence of 0.1% borax. The 0.1-0.2% borax significantly (*p ≤ 0.05*) improved the expressions of *EjNAD-MDH11*. Its maximum expression level was recorded in the loquats treated with 0.2% borax, which was 10.75-fold higher than control. The expression of *EjNAD-MDH12* significantly (*p ≤ 0.05*) reduced under the influence of 0.2-0.3% borax. The *EjNAD-MDH13* was significantly downregulated in fruit pulp of loquat with the foliar application of 0.2% borax (**Figure 8**).

### Correlation analysis

**Figure 9:**
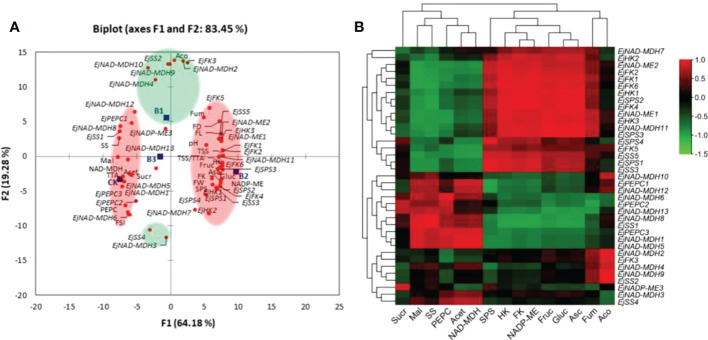
**(A)** Principal component analysis (PCA)among B treatments and sugar-acid attributes of loquat. Clustering of B treatments and measured attributes into groups (coloured circles) is based on their highest squared cosine values corresponding to the factor, F1 (red) or F2 (green). **(B)** Pearson (*n*) correlation analysis between “sugar-acid profile and key enzymes involved in their metabolism” and “relative expressions of related genes” in fruit pulp of loquat. CK, control; B1, 0.1% borax; B2, 0.2% borax; B3, 0.3% borax; FW, fruit weight; FL, fruit length; FD, fruit diameter; FSI, fruit shape index; TSS, soluble solid contents; TTA, total titratable acidity; TSS/TTA, sugar-acid ratio; pH, fruit juice pH; Fruc, fruit fructose; Gluc, fruit glucose; Sucr, fruit sucrose; SPS, sucrose-phosphate synthase; SS, sucrose synthase; HK, Hexokinase; FK, fructokinase.

Principal component analysis (PCA) was conducted to delineate concentration-dependent effects of B on basic fruit quality variables (i.e., fruit weight, size, total soluble solids, total titratable acidity and fruit juice pH), soluble sugars (i.e., fructose, glucose and sucrose), organic acids (i.e., fumaric acid, ascorbic acid, malic acid, *cis*-aconitic acid and acetic acid), key enzymes related to soluble sugars (i.e., SPS, SS, HK and FK) and malic acid metabolism (i.e., PEPC, NADP-ME and NAD-MDH), and relative expression levels of sugar-acid metabolic pathway genes (**Figure 9A**). Based on the highest squared cosine values corresponding to factors F1 or F2, measured attributes were clustered around B treatments. Factor F1, covering 64.18% variability in data (eigenvalue 38.51), showed clustering of fruit weight, fruit length, fruit diameter, total soluble solids, sugar-acid ratio, fruit fructose, fruit glucose, ascorbic acid, activity of SPS, HK, FK, NADP-ME, expression of *EjSPS1-4*, *EjSS3*, *EjHK1-3*, *EjFK1*, *EjFK2*, *EjFK4*, *EjFK6, EjNAD-MDH11*, and *EjNAD-MDH13* with 0.2% borax suggesting its positive influence on these parameters. While, the clustering in opposite quadrant exhibited negative association of 0.2% borax with aforementioned variables. Second factor, covering 19.28% variability in data (eigenvalue 11.566), showed clustering of *cis*-aconitic acid, *EjNAD-MDH2*, *EjNAD-MDH4*, *EjNAD-MDH9*, *EjNAD-MDH10*, *EjSS2*, and *EjFK3* with 0.1% borax. The presence of *EjSS4* and *EjNAD-MDH3* in opposite quadrant indicated the negative association of 0.1% borax with these parameters. Third factor of PCA (not shown), covering 16.55% variability in data (eigenvalue 9.927), showed clustering of pH, fruit sucrose, fumaric acid, *EjNADP-ME*, and *EjNAD-MDH7* with 0.3% borax. Thus, principal component analysis helped to delineate individual roles of B concentrations in regulating sugar-acid metabolism of loquat (**Figure 9A**).

The correlation between “relative expression levels of sugar-acid metabolism-related genes” and “sugar-acid profile and key enzymes related to their metabolism” was analysed (**Figure 9B**). The fructose and glucose were positively correlated with *EjSPS1-4*, *EjSS5*, *EjHK1-3*, *EjFK1*,*2*,*4-6, EjNAD-ME1,2*, and *NAD-MDH7,11*. The fruit sucrose content was negatively correlated with most of the studied genes except *EjSPS4*, *EjPEPC2, EjNAD-ME3*, and *EjNAD-MDH6,12*. The ascorbic acid was significantly (*p ≤ 0.05*) positively correlated with *EjSPS1-3*, *EjSS5*, *EjHK1-3*, *EjFK1*,*2*,*4,6, EjNAD-ME1,2*, and *NAD-MDH7,11*. The fruit malic acid content was significantly (*p ≤ 0.05*) positively correlated with *EjSS1*, *EjPEPC3*, *EjNAD-MDH1*, *EjNAD-MDH5*, *EjNAD-MDH8* and *EjNAD-MDH13*, while significantly (*p ≤ 0.05*) negatively correlated with *EjSPS1-3*, *EjSS5*, *EjHK1,3*, *EjFK1*,*2*,*4, EjNAD-ME1,2*, and *NAD-MDH11*. The negative association of *cis*-aconitic acid was observed with the expression of *EjSS4* and *EjNAD-MDH3*. A positive correlation was also found between acetic acid and *EjPEPC3*, *EjNAD-MDH1* and *EjNAD-MDH5*. The enzymatic activity of SPS was positively correlated with *EjSPS1-4*, *EjSS5*, *EjHK1-3*, *EjFK1*,*2*,*4-6, EjNAD-ME1,2*, and *NAD-MDH7,11*, while SS only found significantly (*p ≤ 0.01*) and positively associated with *EjSS1* and *EjNAD-MDH8*. The HK and FK activity significantly (*p ≤ 0.05*) and positively correlated with *EjSPS1-4*, *EjSS5*, *EjHK1-3*, *EjFK1*,*2*,*4-6, EjNAD-ME1,2*, and *NAD-MDH7,11*. The enzymatic activity of PEPC was positively correlated with *EjSS1*, *EjPEPC2,3* and *EjNAD-MDH1,5,6,8,13*. The NADP-ME activity was found significantly (*p ≤ 0.05*) positively associated with *EjSPS1-3*, *EjHK1*, *EjFK1*,*2*,*4,6* and *NAD-MDH11*, while negatively correlated with *EjSS1* and *EjNAD-MDH8*. The NAD-MDH activity positively correlated with *EjPEPC3*, *EjNAD-MDH1*, *EjNAD-MDH5* and *EjNAD-MDH6* (**Figure 9B**).

## Discussion

### Soluble sugars

The quality of fruit is heavily influenced by soluble sugars and organic acids, the two main components of fruit flavor (Borsani et al., 2009). At the ripe fruit stage, the soluble sugar content of ‘Jiefangzhong’ loquat was increased to a maximum of 19 mg·ml^-1^ in the current investigation. Previous research has shown that the primary sugars that may be found in loquat fruits are sucrose, glucose, and fructose (Toker et al., 2013; Wei et al., 2017). When it comes to the amount of sugar that they contain, various loquat cultivars have varying degrees of variation. The majority of cultivars have a high concentration of sucrose, followed by fructose and glucose, although wild species do not have any sucrose (Liu et al., 2016). According to the findings of our study, the fructose level was found to be the highest among the soluble sugars that were examined.

In the present study, maximum fructose and glucose were recorded in the loquats treated with 0.2% borax. In a previously reported study, foliar spraying of B alone or in combination with Zn led to a large rise in the concentration of non-reducing sugar in fruit juice. On the other hand, these sprays led to a significant reduction in the concentration of reducing sugar and total sugar in pomegranate (Maity et al., 2021). After being sprayed with B, it was found that the content of sugar in papaya, mandarin orange, mango, and pomegranate fruits increased (Babu and Yadav, 2005; Anees et al., 2011; Davarpanah et al., 2016; Subedi et al., 2019). The effect of B on sugar concentration could be attributed to its role in photosynthesis, starch and nucleic acid metabolism, the transport of sugars, and carbohydrate metabolism (Maity et al., 2021).

**Figure 10:**
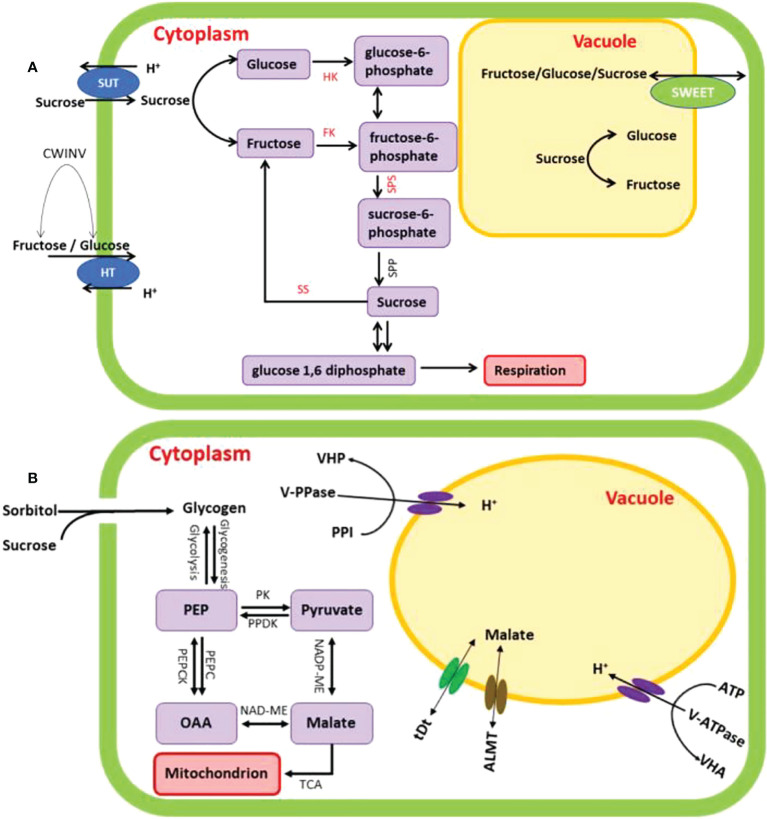
Proposed schematic representation of soluble sugars **(A)** and malic acid **(B)** metabolic pathway in fruit cell. SUT, sucrose transporters; CWINT, cell wall invertase; HT - hexose transporters; HK, hexokinase; FK, fructokinase; SPS, sucrose phosphate synthase; SS, sucrose synthase; SPP, sucrose-phosphate phosphatase; SWEET, sugars will eventually be exported transporter; PK, phosphokinase; PPDK, pyruvate phosphate dikinase; PEPC, phospho*enol*pyruvate carboxylase; NADP-ME, NADP-dependent malic enzyme; NAD-MDH, NAD-malate dehydrogenase; ALMT, aluminium activated malate transporter; tDt, terminal deoxynucleotidyl transferase.

During soluble sugars metabolism in plant cell, sucrose is cleaved into UDG-glucose and fructose by sucrose synthase (SS), or can be cleaved into glucose and fructose by invertases (Ghosh et al., 2013; Irfan et al., 2014; Chen et al., 2017). Fructokinases (FKs) and hexokinases (HKs) can phosphorylate free fructose with high substrate specificity and affinity (Renz and Stitt, 1993; Irfan et al., 2016; 2021; 2022). The cleavage of sucrose and the metabolism of sugar are crucial processes in the formation of healthy vascular tissue, and hence it is thought that fructose phosphorylation by FKs and HKs is required for these processes to take place German et al., 2003; Damari-Weissler et al., 2009; Kumar et al., 2016; 2019; Kumari et al., 2022). Both sucrose phosphate synthase (SPS) and sucrose synthase (SS) are important prerequisites in the biochemical process that results in the formation of sucrose. The synthesis of 6-phosphate sucrose from UDP-glucose and 6-phosphate fructose is facilitated by the presence of SPS (Stein and Granot, 2019), whereas SS converts sucrose into UDP-glucose and fructose (Ruan, 2014) (**Figure 10A**). The majority of the SS proteins may be found in either the cytosol or the plasma membrane, but some can be localized in the vacuole, the cell wall, or the mitochondria (Stein and Granot, 2019). In *Arabidopsis thaliana* and *Malus domestica*, there are 4 and 6 *SPS* genes, respectively (Langenkämper et al., 2002; Li et al., 2012). The number of *SS* genes varies greatly among plant species. There are 6, 8, 12, and 14 *SS* genes in Arabidopsis, carrot (*Daucus carota*), soybean (*Glycine max*) and tobacco (*Nicotiana tabacum*), respectively (Wang et al., 2015; Xu et al., 2019). In Chinese pear, it is reported that there were thirty SS genes (Abdullah et al., 2018).

The expressions of most important genes involved in sugar metabolism and accumulation were studied under the influence of B treatments. The SPS genes showed relative high expression level, while sucrose content was very low. The high transcript level, indicated a negative correlation between sucrose accumulation and SPS activity. A possible reason for this discrepancy is that sucrose is the major soluble sugar in tomato (Dali et al., 1992) and watermelon (Liu et al., 2013; Zhu et al., 2017) but the ‘Jiefangzhong’ loquat mainly showed the accumulation of fructose (Li et al., 2015a).

The enzyme known as sucrose synthase (SS) is also capable of catalyzing the reversible process of the production of sucrose. In peaches and pears, the SS activity has a positive correlation with the amount of sucrose present, but in strawberries and papaya, the correlation is negative (Moriguchi et al., 1992; Lo Bianco et al., 2000; Zhou and Paull, 2001; Basson et al., 2010). It has been hypothesised that when there is a high quantity of glucose and fructose, the SS may be able to be down-regulated in order to lower the enzyme activity (Stein and Granot, 2019). In this study, the *EjSS1-4* showed negative correlation with sucrose content. The *EjSS3,5* showed positive correlation with glucose and fructose. Similar to sucrose, *EjSS2,4* showed negative correlation with glucose and fructose. These results indicated that *EjSS3* or *EjSS5* might were the candidate genes related to fructose and glucose synthesis, while *EjSS2,4* for sucrose degradation at fruit ripening stage.

The glucose and fructose content, activities of their metabolism related enzymes and expressions *EjHK* and *EjFK* genes increased in loquats with the application of B. All *EjHK* and *EjFK* genes exhibited significantly positive correlation with fructose and glucose contents as well as SPS, HK and FK activity of loquat fruits. However, the expression level of *EjFK3* showed negative correlation with glucose and fructose contents, suggesting that *EjFK3* may play a crucial role in regulating the accumulation of glucose through post-transcriptional level (Jiang et al., 2019).

### Organic acids

Aliphatic carboxylic acids, sugar-derived organic acids, and phenolic acids are the three categories that may be used to classify fruit organic acids (Ren et al., 2017). The majority of the organic acids found in loquat fruits are aliphatic carboxylic acids. Some examples of these types of acids are fumaric acid, ascorbic acid, and malic acid (Kumar et al., 2017; Batista-Silva et al., 2018; Sáenz-Galindo et al., 2018). In the present study, the optimized UPLC-MS method was used to detect fumaric acid, ascorbic acid, malic acid, *cis*-aconitic acid and acetic acid from the fruit pulp of loquat under the influence of foliar applied B. Among them, the content of malic acid accounted for about 70%-80% of the total acid. These results are in line with the previous findings about organic acid profile of loquat (2009; Chen et al., 2007; Yang et al., 2021). The composition and content of organic acids in loquat fruits have genetic variation (Famiani et al., 2015), and the differences are also manifested between different varieties (Chen et al., 2009). In present study, the cultivar “Jiefangzhong” was used as plant material which is already reported as high-acid cultivar (Chen et al., 2007; 2009; Ali et al., 2021a; Yang et al., 2021).

Fruits with intermediate acidity tend to be more palatable, but increasing acid content can often lower the quality of the fruit (Zhang et al., 2021). Organic acids build throughout fruit development and are utilised as respiratory substrates as the fruit ripens (Raza et al., 2022). The balance of organic acid production, membrane transit, and breakdown or use determines the ultimate organic acid content in ripened fruits (Sadka et al., 2000; Cercós et al., 2006; Sharma et al., 2022). In this process, malic acid metabolism-related enzymes including phospho*enol*pyruvate carboxylase (PEPC), NADP – dependent malic enzyme (NADP-ME), and NAD-malate dehydrogenase (NAD-MDH) may potentially play a role in fruit malic acid biosynthesis and degradation (Chen et al., 2009; Wu and Chen, 2016). The first step in the production of malic acid begins in the cytosol with phospho*enol*pyruvate (PEP), which is then actively transported into the mitochondria and transformed to oxaloacetate (OAA) by phospho*enol*pyruvate carboxylase (PEPC) (Tayal et al., 2022). Then, NAD-malate dehydrogenase (NAD-MDH) catalyses the condensation of OAA to produce malic acid. The cytosolic enzyme NADP - dependent malic enzyme (NADP-ME) catalyses the conversion of pyruvate to malic acid (Chen et al., 2009; Ma et al., 2019). According to these metabolic routes, malic acid is mostly synthesised by the catalytic activities of PEPC, NADP-ME and NAD-MDH (Wu and Chen, 2016; Zhou et al., 2019) (**Figure 10B**).

Because of the relevance and high quantity of malic acid in fruits, great progress has been made in determining the metabolism of malic acid in fruits. NAD-MDH activity is inversely linked to NADP-ME activity (Chen et al., 2009), indicating that NAD-MDH and NADP-ME may both play essential roles in malate production and degradation, respectively. Yang et al. (Yang et al., 2011) also cloned the genes encoding *EjPEPC*, *EjNADP-ME*, and *EjcyNAD-MDH*, and they discovered that the transcript level of *EjNADP-ME* in the high-acid cultivar was considerably greater than that in the low-acid cultivar, but *EjNADP-ME* and *EjmNAD-MDH* expression patterns were comparable in both cultivars, however *EjPEPC* and *EjcyNAD-MAD* expression patterns were different, suggesting that the expression of these genes may be crucial in controlling malic acid production in loquat fruit.

Due to coenzyme specificity, subcellular localization, and biochemical function, NAD-malic enzyme (NAD-ME) contributes 70-80% to malic acid accumulation (Rao and Dixon, 2016). In a recent study, it has been proved that *NAD-cytMDH* is a key gene that regulates the acidity of peach fruits (Etienne et al., 2002). The overexpression of *MdNAD-ME* genes significantly increased the content of malic acid in apple callus (Drincovich et al., 2001). It has been reported earlier that NADP-ME catalyzes the carboxylation of pyruvate and fixes CO_2_ to produce malic acid in grapes (Sweetman et al., 2009). NADP-ME plays a major role in the degradation of malic acid in the cytoplasm, such as the content of organic acids in apple were negatively correlated with NADP-ME activity (Yao et al., 2009). The change in the activity of NAD-ME is consistent with the biosynthesis of malic acid, the increase in fruit respiration, and the gradual decrease of malic acid during fruit ripening (Khan et al., 2018). During storage of climacteric fruits e.g., apple, pear, banana, etc. the respiration increases, accompanied by accelerated degradation of malic acid (Farrar et al., 2000; Ruan, 2014). In the current study, the fruit malic acid content was significantly and positively correlated with *EjSS1*, *EjPEPC3*, *EjNAD-MDH1*, *EjNAD-MDH5*, *EjNAD-MDH8* and *EjNAD-MDH13*, while significantly (*p ≤ 0.05*) negatively correlated with *EjSPS1-3*, *EjSS5*, *EjHK1,3*, *EjFK1*,*2*,*4, EjNAD-ME1,2*, and *NAD-MDH11*.

## Conclusions

The results of the current study suggest that fructose and malic acid are the predominant sugar and acid in fruit pulp of loquat, respectively. Among B treatments, 0.2% borax was the promising treatment to enhance soluble sugars and reduce malic acid concentration in fruit pulp of loquat. Boron treatments remarkably improved the soluble sugars content by regulating the activities of SPS, SS, HK and FK enzymes, and altering the expressions of related genes. The combined activity of many enzymes, including PEPC, NADP-ME, and NAD-MDH, was responsible for controlling the dynamics of the malic acid concentrations evaluated in the present investigation. Correlation analysis suggested that NAD-MDH played a vital role in the decrease of malic acid. These findings not only elucidated previously unknown aspects of the metabolism of soluble sugars and organic acids, but they also provide a significant resource for prospective studies on the application of molecular breeding techniques to loquat fruit.

## Data availability statement

The original contributions presented in the study are included in the article/**Supplementary Material**. Further inquiries can be directed to the corresponding authors.

## Author contributions

Conceptualization, MA, YH, and FC; Data curation, MA and RA; Funding acquisition, YH; Methodology, MA, AY, and FC; Supervision, YH and FC; Writing – original draft, MA; Writing – review & editing, RA, RR, SEj, SA, SEr, YH, and FC. All authors contributed to the article and approved the submitted version.

## Funding

This research was funded by Ministerial and Provincial Joint Innovation Centre for Safety Production of Cross-Strait Crops, Fujian Agriculture and Forestry University, Fuzhou 350002, China.

## Conflict of interest

The authors declare that the research was conducted in the absence of any commercial or financial relationships that could be construed as a potential conflict of interest.

## Publisher’s note

All claims expressed in this article are solely those of the authors and do not necessarily represent those of their affiliated organizations, or those of the publisher, the editors and the reviewers. Any product that may be evaluated in this article, or claim that may be made by its manufacturer, is not guaranteed or endorsed by the publisher.
