# Supplementary material for: Sugar and acid profile of loquat (Eriobotrya japonica Lindl.), enzymes assay and expression profiling of their metabolism-related genes as influenced by exogenously applied boron
Source: Front Plant Sci. 2022 Oct 20;13:1039360. doi: 10.3389/fpls.2022.1039360 (PMC9632665; doi:10.3389/fpls.2022.1039360)
Supplement: Supplementary file 1 [file DataSheet_1.docx]

Sugar and Acid Profile of Loquat (*Eriobotrya japonica* Lindl.), Enzymes Assay and Expression Profiling of Their Metabolism-Related Genes as Influenced by Exogenously Applied Boron

Muhammad Moaaz Ali^1,2^, Raheel Anwar^3^, Rana Naveed Ur Rehman^4^, Shaghef Ejaz^5^, Sajid Ali^5^, Ahmed F. Yousef^6^, Sezai Ercisli^7^, Xiaobo Hu^2^, Youming Hou^1,*^ and Faxing Chen^2,*^

^1^State Key Laboratory of Ecological Pest Control for Fujian and Taiwan Crops, College of Plant Protection, Fujian Agriculture and Forestry University, Fuzhou, Fujian 350002, China

^2^Institute of Subtropical Fruits, Fujian Agriculture and Forestry University, Fuzhou 350002, China

^3^Institute of Horticultural Sciences, University of Agriculture, Faisalabad 38000, Pakistan

^4^Department of Horticulture, Faculty of Food and Crop Science, PMAS-Arid Agriculture University, Rawalpindi 46300, Pakistan

^5^Department of Horticulture, Faculty of Agricultural Sciences and Technology, Bahauddin Zakariya University, Multan 66000, Pakistan

^6^Department of Horticulture, College of Agriculture, University of Al-Azhar (Branch Assiut), Assiut 71524, Egypt

^7^Department of Horticulture, Agricultural Faculty, Ataturk University, 25240 Erzurum, Turkey

*** Correspondence:**Youming Hou and Faxing Chen
ymhou@fafu.edu.cn (Y.H.); fxchen@fafu.edu.cn (F.C.)

Supplementary Material

#


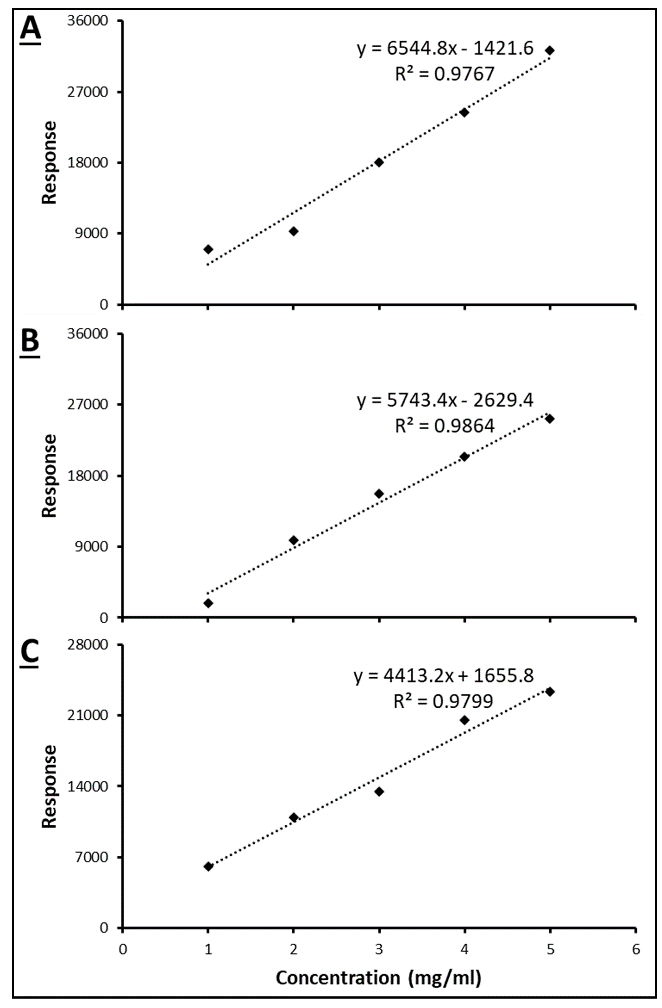


Figure S1. Standard curves for the quantification of fructose (A), glucose (B) and sucrose (C) through HPLC-MS.


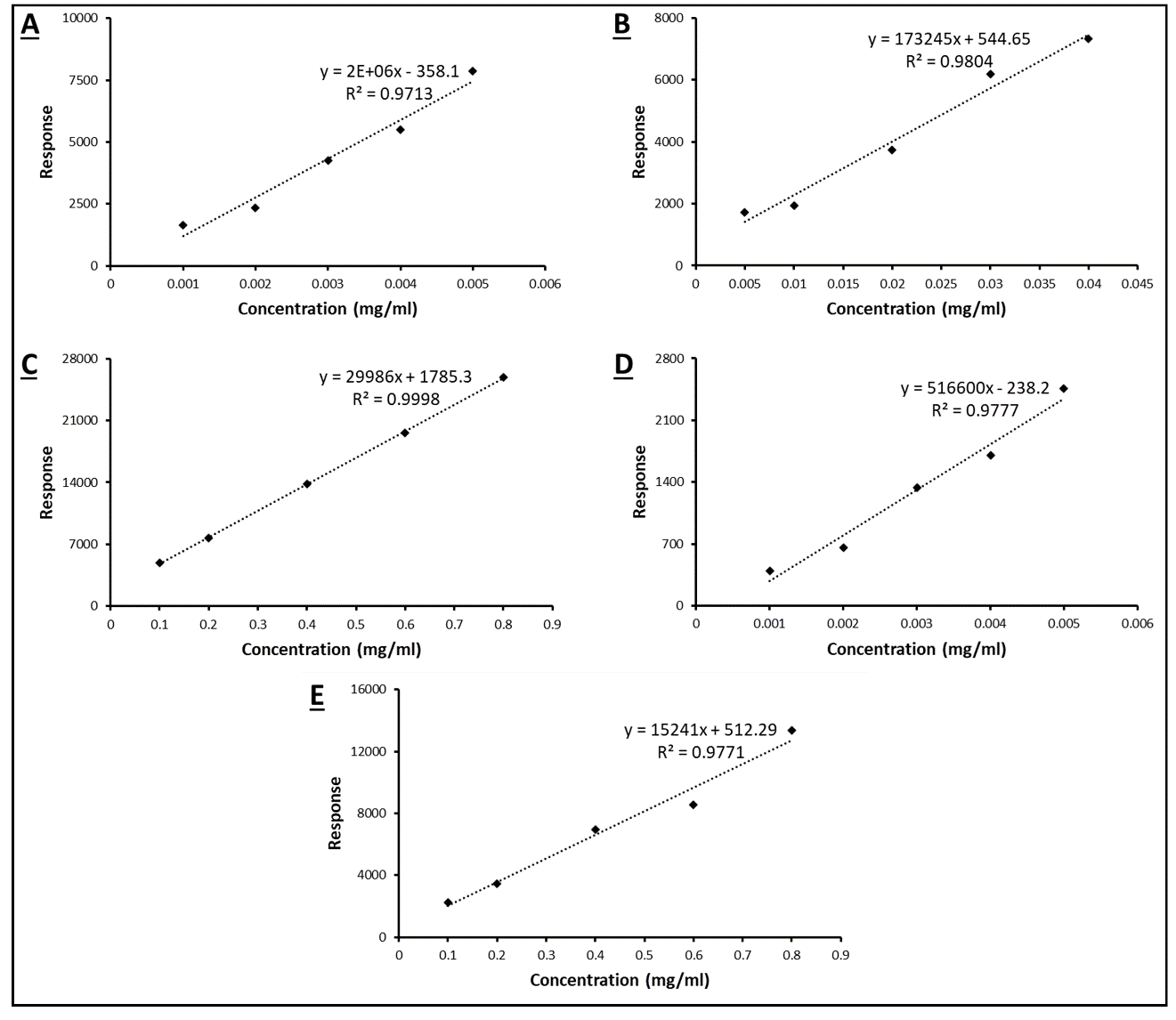


Figure S2. Standard curves for the quantification of fumaric acid (A), ascorbic acid (B), malic acid (C), *cis*-aconitic acid (D) and acetic acid (E) through UPLC-MS.

# Table S1. Sequences of Primer Pairs for qRT-PCR Analysis.

| Code | Gene ID | Forward primer (5' -3') | Reverse primer (5' -3') |
| --- | --- | --- | --- |
| *EjSPS1* | EVM0006877.1 | CTGGTGGCCAGGTCAAGTAT | TCTCCCCAAGATCCTCCTTT |
| *EjSPS2* | EVM0039966.1 | GTTTGCTTGTGGACCCTCAT | GTTTGCTTGTGGACCCTCAT |
| *EjSPS3* | EVM0004626.1 | GGGTCGCTAATTTCAACGAA | ACGGAGAGCCTGAATTCTCA |
| *EjSPS4* | EVM0036648.1 | GCTTTAAACGTGCCAATGGT | AGAATCCAATCCCAGCTCCT |
| *EjSS1* | EVM0040118.2 | TGGATATCATACCCCGCATT | CTCGGCGTATGTCTCTAGGC |
| *EjSS2* | EVM0022638.1 | GGAAATGGGGTTCAATTCCT | TTGGCGGGAACTTAGAAAGA |
| *EjSS3* | EVM0016765.1 | CAATTTCTCAATCGCCACCT | GCTCAAAACCCATTCCTTGA |
| *EjSS4* | EVM0036394.1 | GGTTGAGTGGTACGGGAAGA | TCAGCAATGCAACGGTAGAG |
| *EjSS5* | EVM0016679.1 | GGCAGTTGAGAGATGTGCAA | GCTAAGGTTGGTGCTGCTTC |
| *EjHK1* | EVM0024274.1 | GAACGTGCACATGCAATACC | TCTGATCACCACGGTTCAAA |
| *EjHK2* | EVM0032037.1 | TTTGCGTGTTACAGCTTTGG | AGAAGACCATGCCATTTTGG |
| *EjHK3* | EVM0017610.1 | AGGATGCTTGGAGAAAGCAA | ATTTCCCCATTCCATGTTGA |
| *EjFK1* | EVM0043405.1 | TTGGTGGCTCTTCAGCTTTT | ACTCACGTTCCCCATCACTC |
| *EjFK2* | EVM0036698.1 | TGGCGTCTCTTCCTTTCACT | TCCTCATCTTCAACGGAACC |
| *EjFK3* | EVM0027365.1 | CTTCGAGGTCAAAGCCGTAG | GCTTCAGACTCGTTGGGAAG |
| *EjFK4* | EVM0013286.1 | AGGGAGTGTGCGTGGATAAC | CAAAACGCCAGCTTCCTTAG |
| *EjFK5* | EVM0022899.1 | TGTTGCAGTTGGCATAGCTC | ACTCACGTTCCCCATCACTC |
| *EjFK6* | EVM0022143.1 | AGCTGATGGAGAGCGTGAAT | TTTTCATTGCAGCAAGATGG |
| *EjPEPC1* | EVM0016068.1 | CCAAGCATCTGGAAATTGGT | CGAGCACATCAGATGGAGAA |
| *EjPEPC2* | EVM0022212.1 | TGGAGCCTCTCGAACTTTGT | CATTCTTGCCTACGCTCCTC |
| *EjPEPC3* | EVM0021989.1 | TTTGCAGAAAGATGCACGAC | CCGAATATCCAACCATCACC |
| *EjNAD-ME1* | EVM0043159.1 | GTCGTCAGACACCTCAAGCA | ACGTCCCTTTGTTGAACCAG |
| *EjNAD-ME2* | EVM0026642.1 | CCAACCATGAATGCTGAATG | CCGATCCCTGGAAACAGATA |
| *EjNADP-ME* | EVM0008119.1 | GCAAAGGAGAGCAACTGGAC | AGCCCTGCTAAGACCACAGA |
| *EjNAD-MDH1* | EVM0027402.1 | CATTTGGACCCAGCAAATCT | GACCAAGGGGTTCATCTTCA |
| *EjNAD-MDH2* | EVM0031471.1 | CTCACGGGCATAGACCTTGT | TGCTGCAATAGGAACTGTCG |
| *EjNAD-MDH3* | EVM0029796.1 | TCGCTCTCTTTCGGAACATT | TCCTGAGCATTGACATCGAG |
| *EjNAD-MDH4* | EVM0038150.1 | GAGGGACCAGAGGAAGATCC | TCTGTGCCTCCTCATCAGTG |
| *EjNAD-MDH5* | EVM0031615.1 | TGTAGAGGCATGCACTGGAG | AACCAAAACCTTGCAGTTGG |
| *EjNAD-MDH6* | EVM0006816.1 | TGAAGGAATTTGCACCATCA | GTTGCATGGTTGACATCAGG |
| *EjNAD-MDH7* | EVM0014238.1 | CATCTGGGGCAATCACTCTT | GAGTCCCAAGAACCCAATCA |
| *EjNAD-MDH8* | EVM0030403.1 | GCTACTCATCCGCCTCTGTC | CTGGTGTTGATGTGGCTGAC |
| *EjNAD-MDH9* | EVM0041440.1 | AAGGGATCTGCCACATTGTC | ACCCCAAAACTTCCTCGACT |
| *EjNAD-MDH10* | EVM0029104.1 | CCCCTGCTATCCAAGACAAA | CGGTCAGATCAGACGCTACA |
| *EjNAD-MDH11* | EVM0017505.2 | ACCTGTCAGGGAACTTGTGG | ACACCCATTGAAACCCATGT |
| *EjNAD-MDH12* | EVM0022235.1 | GCCAGACCAACCTATTGCAT | CCAACAAGCTTGCTCTTTCC |
| *EjNAD-MDH13* | EVM0043622.1 | GAGCATTGGCATTGATCCTT | TGCAGGTATGTTTGGTGCAT |
| *EjAct* | EVM0004523.1 | GGAGCGTGGATATTCCTTCA | GCTGCTTCCATTCCAATCAT |

The gene sequences were retrieved from loquat genome available at GigaScience Database (<http://gigadb.org/dataset/view/id/100711>) (Jiang et al., 2020).=

Jiang, S., An, H., Xu, F., and Zhang, X. (2020). Chromosome-level genome assembly and annotation of the loquat (*Eriobotrya japonica*) genome. *Gigascience* 9. doi: 10.1093/gigascience/giaa015.
